# Nutritional Assessment and Management in Paediatric Chronic Kidney Disease

**DOI:** 10.1155/2021/8283471

**Published:** 2021-10-12

**Authors:** Jumanah Ziyad Azzouz, Osama Yousef Safdar, Farah Idriss Awaleh, Alya Abdullah Khoja, Ali Alawi Alattas, Abdulkarim Abbas Jawhari

**Affiliations:** ^1^King Abdulaziz University Hospital, Jeddah, Saudi Arabia; ^2^Center of Excellence in Pediatric Nephrology, King Abdulaziz University, Jeddah, Saudi Arabia

## Abstract

Nutrition in paediatrics has always been one of the most important factors for optimal growth. Children with chronic kidney disease (CKD) need special consideration for better long-term outcomes, including nutritional status, optimal height, and cognitive function. Nonetheless, there are many obstacles to overcome to attain optimal linear growth and nutritional status in children with CKD. This review highlights the need for tools to assess the growth parameters in CKD. In addition, recommendations for dietary intake play a major role in controlling electrolyte disturbances in patients with CKD. For example, it is still unclear whether it is better to restrict phosphate sources in inorganic, organic, or food additives. The review also summarises different factors such as fluid intake, route of feeding, and essential nutrients that require particular attention in paediatric patients with CKD. In summary, a multidisciplinary team is needed to devise individual nutritional plans to achieve the best outcome and improve the quality of life of patients.

## 1. Introduction

Nutrition is crucial for the growth and neurocognitive development of children [1]. Children with chronic kidney disease (CKD) require specific nutritional care because their renal function is compromised, and they cannot absorb or excrete some essential nutrients to achieve normal growth and metabolic balance [2, 3]. CKD is defined as an “irreversible insufficiency of renal function that gradually progresses to end-stage renal disease” [4]. Irreversible kidney insufficiency is characterised by a glomerular filtration rate (GFR) of less than 90%, while end-stage renal disease (ESRD) occurs when dialysis or renal replacement therapy is necessary for life [1]. Procedures such as haemodialysis are associated with protein loss, salt wasting, and disruption of the hydration state [5]. Malnutrition in patients with CKD has been addressed in multiple studies [1, 5]. Different guidelines are available to assess and manage the daily nutrient requirements in children with CKD, including the Canadian, American [6], Australian [7], and European [8] guidelines. Specific nutritional elements, including protein, sodium, potassium, and phosphorus, require special consideration in these patients [9–11], and fluid intake also demands strict control [12, 13]. Assessing nutritional status and adequate diet is relatively difficult, especially in children [1], and no established nutritional measures can be used in all children [1, 3]. However, the World Health Organization (WHO) states that values of two standard deviations (SDs) below the national growth chart for sex-specific medians of weight-for-height, weight-for-age, and height-for-age are the limits separating well-nourished and malnourished children [11, 14, 15]. A study reported that 50% of all paediatric patients with ESRD end up with a final height below the 3^rd^ percentile [16]. Unexpectedly, new studies are now directing attention to obesity, which can also be observed in children with CKD [1].

This review discusses the tools used to assess growth parameters, recommendations for dietary intake, and other factors such as fluid intake, route of feeding, and essential nutrients that require particular attention in paediatric patients with CKD. Its significance is strengthened by the higher prevalence of CKD in children (18.5–58.3 cases/million with stage 2 CKD) than in adults [1, 3].

## 2. Methodology

In this review article, we aim to review the parameters of nutrition and growth in paediatric chronic kidney disease and discuss the most recent evidence-based methods for evaluation of growth in CKD patients. We reviewed studies pertaining to this topic by searching for relevant studies on a variety of databases such as PubMed using specific keywords chronic kidney disease, paediatrics, kidney failure, nutrition, and growth. The articles found were screened to ensure their relevance to the topic of research. A total of 81 articles will be summarized in this review.

### 2.1. Growth Parameters

Growth parameters in children with CKD need monitoring at least twice as frequently as in healthy children of the same age. Anthropometric monitoring of infants with stages 2–4 CKD is recommended every 2–6 weeks. For children aged 1–3 years, monitoring is recommended every 1–3 months. Children older than 3 years of age should be monitored every 1–6 months, depending on the degree of CKD. Patients undergoing chronic dialysis require monthly nutritional evaluations [17, 18]. For infants and children up to 2 years of age, it is recommended to monitor weight, length, weight-for-length, and head circumference z-scores using the WHO growth chart. Patients over 2 years of age can be evaluated by monitoring their weight, height, BMI, and growth velocity using the CDC 2–20 year growth charts [19]. For every decrease in height by one SD, mortality increases by 14% [20]. The multicentre Chronic Kidney Disease in Children (CKiD) study examined short stature in children with CKD and found that those who gained height had better health and quality of life [21]. Another study suggested that children with glomerular causes of CKD require repeated monitoring for protein-energy wasting because they tend to have notable weight loss compared with those with nonglomerular CKD [22]. Monitoring of nutritional status can be achieved using biochemical parameters, including serum albumin, prealbumin, total protein, transferrin, and creatinine levels, as well as haemoglobin, total lymphocyte counts, cholesterol, triglycerides, and retinol binding protein. Although all of these may contribute to a comprehensive nutritional assessment, none of them can be considered sensitive or specific enough to be used as a diagnostic marker for protein-energy wasting. Serum albumin has been extensively studied, particularly in adults with CKD, but there is indisputable evidence against its use as an exclusive marker of nutritional status [23–25]. Growth hormone has significant benefits in increasing height and catch-up growth in children with CKD because of its important role in stimulating linear growth, increasing muscle mass, and improving bone density. Moreover, optimising height by using growth hormone was found to be associated with improved physical and social functioning in children with CKD [21, 26, 27].

### 2.2. Caloric Intake

Patients with CKD need the same caloric intake as healthy children of the same chronological age. The energy requirements for each child are determined by body size, physical activity, and other attributes. The need for adequate nutrition is crucial during the first 2 years of life to optimise development and growth. For example, infants should receive, if feasible, breast milk (which may require caloric supplements) or an age-adjusted formula. Since many infants with more advanced CKD require potassium and/or phosphorous restriction, formulas that are low in these ingredients may be required [16]. Numerous specialised formulas are available to provide adequate dietary intake for infants and children with CKD. Some patients also have increased caloric density, and it is useful in patients with oliguric CKD to provide a more concentrated form of nutrition, with the assistance of an experienced renal dietician, to meet their specific caloric, solute, electrolyte, and metabolic needs [28]. For older children, additional supplements may be necessary to attain the appropriate energy requirements [29]. In the nondialysed CKD population, energy intake should be supplemented to provide approximately 100% of the daily estimated energy expenditure (for chronological age, activity level, and body size) [18]. Similarly, protein intake should be at least 100% of the daily recommended intake (DRI) for age and body size, with some patients with advanced CKD on dialysis requiring up to 140% of the DRI for protein. However, certain factors such as uraemia, inflammation, and comorbidities affect the estimated energy needs of children with CKD. These barriers affect the ability to receive the appropriate caloric intake and require continuous monitoring of growth and related dietary adjustments [30].

### 2.3. Route of Feeding

There are numerous reasons why children with CKD do not achieve appropriate nutrient intake. In some patients, oral feeding may be suboptimal, which may affect appetite and increase gastrointestinal symptoms, creating challenges for meeting caloric needs. Patients with CKD may need further supplementation or manipulation of enteral feeding by the nasogastric (NG) tube or gastrostomy tube (G-tube), and studies have shown that nutritional supplementation with tube feeding more readily results in an increase in weight and BMI, but not necessarily in height; however, about 50% of patients become overweight or obese [31]. Regardless of the modality, tube feeding is designed to provide the required fluid, caloric, and protein intake that cannot be achieved by oral feeding alone. Moreover, tube feeding may allow better medication tolerance [16]. While many children are initially started on NG tube feeds, there is clear evidence that G-tube feeds are generally more effective. Data from the International Paediatric Peritoneal Dialysis Network (IPPN) [32] strongly show the superiority of G-tube to NG tube feeding in some children with ESRD. However, each modality is associated with risks and complications. There is an increased risk of infection with G-tubes, especially in patients receiving peritoneal dialysis [33, 34]. The major disadvantages of NG feeding include the aesthetic appearance of the tube, the requirement to periodically change it, and the increased risk of developing gastroesophageal reflux [31]. As shown by Yoo et al. [35], fundoplication may be necessary in children with moderate-to-severe gastroesophageal reflux disease who have persistent vomiting to optimize tube feeding. Finally, it is important to establish that the long-term goal of tube feeding is to achieve full oral feeding posttransplant. While the infant is reliant on tube feeding and during frequent hospitalisations, parents should be supported to maintain their infant's oral motor skills as much as possible [36].

### 2.4. Fluid Intake

Fluid intake in patients with CKD, especially those on dialysis, should be carefully monitored because intravascular fluid imbalance can lead to cardiovascular complications, which will directly increase morbidity and mortality risks [37, 38]. While fluid overload is harmful, aggressive fluid restriction may be toxic to the child's myocardium [39, 40]. In all cases, fluid intake must be balanced and carefully assessed and managed.

Nowadays, the assessment of fluid status depends on regular physical examinations in accordance with predialysis and intradialytic blood pressure and weight gain. Nevertheless, many studies have suggested that these methods are insufficient for adjusting the target weight gain. Other methods, such as bioimpedance spectroscopy, are used to estimate total body water, lean and adipose mass, tissue extracellular water, and overhydration; inferior vena cava collapsibility index; relative blood volume monitoring; N-terminal probrain natriuretic peptide; and lung ultrasound were all shown to have limitations, mostly through single-centre studies. Fluid overload has mostly been tested in specific clinical settings [41–49].

Patients with CKD and comorbidities have a higher potential to be “overhydrated” [50]. Many studies have reported no significant difference between isotonic or hypotonic intravenous fluid administration in managing these patients [50, 51]. However, some studies have suggested that isotonic fluids lead to lower risk of hyponatremia [52]. Adjusting the amount of sodium consumed through diet or medication is the main step in managing fluid status in patients with CKD [41].

### 2.5. Protein

The protein requirements of children with CKD are very specific, given the impact of protein intake on mortality. Therefore, protein intake is often limited in these patients. However, children with CKD who have reached the point of needing dialysis will have increased protein needs due to losses during haemodialysis and peritoneal dialysis. Protein losses are twice as large per square meter of the body surface area in infants on peritoneal dialysis than in adolescent patients who are comparable to adults in size [9]. The current Kidney Disease Outcomes Quality Initiative (KDOQI) guidelines [6, 18] recommend supplying children with stages 2-3 CKD with 100–140% of the DRI of protein for ideal bodyweight, while children with more advanced CKD should receive 100–120% of the DRI [16]. To achieve appropriate growth, children need to be kept in a positive nitrogen balance by assessing protein and caloric intake. Low-protein diets have not been recommended to reduce the progression of renal disease because there is no conclusive evidence that long-term protein restriction is effective against CKD progression [53]. However, increased protein consumption in children with CKD may increase the accumulation of nitrogenous waste products and uraemia. During the immediate posttransplant period, protein needs are increased by approximately 50% in association with surgical stress and the catabolic effects of steroids and return to the normal recommendations (DRI for age) approximately 3 months after transplantation [53].

### 2.6. Micronutrients: Vitamins and Minerals

Most patients with CKD undergoing dialysis have abnormal renal metabolism, insufficient intake, decreased gastrointestinal absorption, metabolic imbalances, and dialysis-related losses, leading to increased risk of vitamin and mineral deficiencies [5, 54].

The KDOQI recommends that children with CKD on dialysis receive sufficient vitamin A, B, C, E, and K, as well as minerals including copper and zinc. These micronutrients should be given as supplements if the dietary intake is not sufficient to achieve DRI. Water-soluble vitamins, including thiamine (B1), pyridoxine (B6), folate (B9), and vitamin C (ascorbic acid), are the most frequent deficiencies, and patients should also receive water-soluble vitamin supplements [5, 18, 54].

Vitamin D plays a major role in the bone and mineral disorders observed in patients with CKD. A cohort study in children with CKD reported a 28% prevalence of 25-hydroxyvitamin D deficiency, with increased risk of secondary hyperparathyroidism and decreased serum levels of 1,25(OH)2D. 25OHD deficiency is common in children with CKD; however, it is a modifiable risk factor that can be corrected through milk intake and vitamin D supplementation [55].

Canepa et al. estimated a 40% prevalence of hyperhomocysteinemia in children with CKD. Folic acid and vitamin B12 levels were found to be low in a small percentage of children with hyperhomocysteinemia [56]. However, they are considered part of the standard water-soluble vitamin supplementation in children with CKD [16, 57].

Most children with CKD have anaemia with multifactorial aetiology, but mostly attributable to decreased erythropoietin production. In addition, these patients are prone to iron deficiency. Oral or intravenous iron supplementation should be administered to patients on erythropoietin-stimulating agents to prevent iron storage depletion and preserve target haemoglobin concentrations [16, 58]. Moreover, for the treatment of anaemia in CKD, some children need the previously mentioned vitamin B12 and folic acid supplementation in addition to iron supplementation [58].

Trace minerals making up less than 0.01% of the total human bodyweight, including zinc, selenium, folic acid, pyridoxine, and ascorbic acid, are lost during haemodialysis, as normal renal function is required for mineral homeostasis [59, 60].

As the evidence to support the routine use of vitamins in patients undergoing haemodialysis from systematic analyses is insufficient, an individualised approach to decision-making is recommended [61].

### 2.7. Salt

Sodium requirements directly depend on the cause of CKD, whether it is sodium waste, such as in congenital urinary tract anomalies, the most common cause of CKD [62], or sodium nonwasting glomerular diseases [58], and on urinary output. For patients needing sodium restriction, the KDOQI recommends less than 1500–2400 mg/day [18] to ensure that the growth process is not interrupted [63]. Nguyen et al. proposed many solutions to help monitor sodium levels, including prioritising fresh over refined food [58]. In contrast, in sodium-wasting disease, the loss of sodium is greater than the intake via breastfeeding, formula, or food due to impaired sodium reabsorption. Therefore, sodium supplementation should at least initially start with the age-adjusted DRI; adjustments can then be made to prevent sodium depletion, which would cause growth retardation and hypertension [29, 53, 62, 63]. Although a low-sodium diet shows beneficial effects in nondialysed patients with CKD, adherence to this diet could be challenging, and more studies discussing this matter are needed [64].

### 2.8. Potassium

Potassium also plays a major role in many important cellular functions, such as muscle contraction and nerve conduction [29]. It easily moves from the intra to the extracellular compartment to maintain normal potassium levels (3.5–5 mmol/L), and even a small shift may change the potassium concentration. Therefore, patients with CKD are at risk of both hypokalemia and hyperkalemia, depending on many factors. Patients with mild-to-moderate hypokalemia tend to be asymptomatic compared with those with severe hypokalemia who can experience weakness, cramps, and even paralysis. In contrast, decreased GFR, urinary obstruction, and treatments with potassium-sparing agents would be risk factors for hyperkalemia, which has more undesirable cardiac effects including arrhythmias and cardiac arrest [16, 65, 66]. The KDOQI recommends restricting potassium in infants and young children to 40–120 mg/kg/day and to 30–40 mg/kg/day in older children. It is also important to educate the families of patients at high risk of hyperkalemia about foods containing high or low levels of potassium and potassium binders. [18, 58].

Breastfeeding, using low-potassium formulas, and avoiding high-potassium vegetables and fruits are among the solutions discussed by Nguyen et al. [58, 67]. Foods containing high levels of potassium such as bananas, kiwis, oranges, avocados, potatoes, and tomatoes should be replaced with low-sodium foods such as apples, berries, lemons, carrots, cucumbers, and lettuce [68]. However, the common recommendation to restrict potassium in paediatric patients with CKD needs more evidence [67]. Most observational studies aiming to find associations between cardiac complications and potassium levels focused on adults, and more paediatric studies are needed.

### 2.9. Phosphate

Generally, phosphate levels are elevated in CKD. Hyperphosphataemia and other factors play major roles in the development of cardiovascular events. Therefore, the general approach to manage hyperphosphataemia is to restrict phosphate intake, which should, however, be adequate for normal bone mineralisation and to prevent mineral and bone disorders [69–71].

Normal serum phosphate levels vary with age. For example, in the first 6 months of life, phosphate intake should be 5.2–8.4 mg/dL, and the recommended range decreases with age in normal individuals. In CKD, serum phosphate levels are above the normal range, and phosphate restriction is needed. The KDOQI guidelines recommend limiting phosphorus to 100% of age-related DRI, unless both phosphate and parathyroid hormone are secondarily elevated, in which case the patient should receive 80% of the DRI. [18].

The management of hyperphosphataemia depends on controlling phosphate sources, which are classified into organic (natural food) or inorganic (food additives), with the latter having much higher absorption rates, reaching 90% [72, 73]. High phosphorus content is found in bran cereal, dairy products, and processed meat. In contrast, unprocessed meat, rice cereal, breast milk, fresh fruits, and vegetables contain lower amounts of phosphorus [68]. However, hidden food additives are problematic, and further precautions are needed. [74] Other management methods include the use of calcium-based and noncalcium-based phosphorus binders, especially in patients with advanced CKD [75, 76]. Additionally, when parents are educated about the sources of phosphate and how to avoid them, a reasonable decline in phosphate levels has been observed, compared with noneducated parents [77, 78]. In fact, phosphate levels showed a 25% decline from 1995 to 2010, with positive impacts on patients [79].

### 2.10. Calcium

Calcium, like other micronutrients, should be sufficient to meet the demands of the patient's body. In advanced CKD, calcitriol production decreases, which may affect the intestinal absorption of calcium, causing hypocalcaemia and secondary hyperparathyroidism. Therefore, the KDOQI recommends that the child should receive up to 100–200% of the age-appropriate DRI with a maximum dose of 2500 mg/day, including from food containing calcium, medications such as phosphate binders, and calcium supplementation [18]. However, the amount of calcium absorbed from phosphate or calcium binders is inaccurately known, and more studies are needed to carefully assess calcium intake [80].

In infants, all calcium intake derives from breast milk or infant formula [81]. Later, milk and milk products continue to account for 44–70% of the total calcium intake. Cereal products come after milk as the most relevant sources of calcium. Calcium supplementation comes in many forms and is mandatory for those who do not meet the DRI. Supplementation with carbonate and calcium gluconate is more effective than calcium citrate and calcium chloride, which may cause aluminium toxicity and metabolic acidosis, respectively [16, 18, 29]. In managing secondary hyperparathyroidism, paricalcitol and calcitriol have similar effects, according to a recent meta-analysis [82].

### 2.11. Limitations

The main limitation of this study lies in the lack of new research studies regarding nutritional assessment in CKD patients especially in paediatrics and the new guidelines only focusing on the adulthood age group. Less studies were published regarding the subject in the past few years. We did not capture as much information of outcomes in patients with specified diet and their independent results and how it affected growth in CKD paediatric patients. Another limitation is that most studies focus on a singular nutrient rather than assessing all nutrients and fluid that may affect the growth of a child with CKD.

## 3. Conclusion

Nutritional assessments are complex and critical in paediatric patients with CKD. Many factors should be taken into consideration, starting with family education about food and snack preparation as well as about any expected changes and red flags in their child's status.

### 3.1. Recommendation

We recommend in-depth exploration and studying of malnutrition and inadequate linear growth in children with CKD and the effect of nutrients in different patients. In addition, systemic review with meta-analysis is required to update the clinical practice in paediatrics CKD patients. Moreover, it is important to optimize the care process to allow each individual patient to obtain the greatest possible benefit through the assistance of a multidisciplinary team including doctors, dialysis team nurses, dietitians, social workers, teachers, and psychologists to fulfil dietary targets and improve life satisfaction.

## Data Availability

This a review of article, and the data are included within the article.

## Additional Points

The nutritional status of children with chronic kidney disease requires special consideration to obtain the best possible outcomes in terms of growth, cognitive function, and quality of life by the multidisciplinary team. Various nutrients and pinpoint areas to control the intake were highlighted.
